# A new hyperspectral image classification method based on spatial-spectral features

**DOI:** 10.1038/s41598-022-05422-5

**Published:** 2022-01-27

**Authors:** Qu Shenming, Li Xiang, Gan Zhihua

**Affiliations:** 1 School of Software, Henan University, Kaifeng, 475001 Henan China; 2 Institute of Intelligence Networks System, Henan University, Kaifeng, 475001 Henan China; 3 International Institute of Intelligent Information Processing, Henan University, Kaifeng, 475001 Henan China

**Keywords:** Computational science, Computer science, Software, Applied optics, Optical techniques

## Abstract

In recent years, more and more deep learning frameworks are being applied to hyperspectral image classification tasks and have achieved great results. However, the existing network models have higher model complexity and require more time consumption. Traditional hyperspectral image classification methods tend to ignore the correlation between local spatial features. In this paper, a new hyperspectral image classification method is proposed, which combines two-dimensional Gabor filter with random patch convolution (GRPC) feature extraction to obtain spatial-spectral feature information. The method firstly performs dimensionality reduction through principal component analysis and linear discriminant analysis and extracts the edge texture and spatial information of the image using a Gabor filter for the reduced-dimensional image. Next, the extracted information is convolved with random patches to extract spectral features. Finally, the spatial features and multi-level spectral features are fused to classify the images using the Support Vector Machine classifier. In order to verify the performance of this method, experiments were conducted on three widely used datasets of Indian Pines, Pavia University and Kennedy Space Center. The overall classification accuracy reached 98.09%, 99.64% and 96.53%, which are all higher than other comparison methods. The experimental results reveal the superiority of the proposed method in classification accuracy.

## Introduction

In recent years, with the development of remote sensing technology, multi-source remote sensing imaging means such as hyperspectral, infrared, and radar play an increasingly important role in important fields such as precision agriculture, resource investigation, environmental monitoring, national defense and military^1,2^. However, hyperspectral images (HSIs) is a three-dimensional data cube with special high dimensionality, strong correlation between adjacent bands and highly nonlinear data structure and few training samples, and it makes the classification of HSI with many difficulties^3^.

How to effectively overcome the above problems, extract multi-level nonlinear discriminant features from HSI data, and improve the classification accuracy of the model, researchers have proposed many methods^4,5^. For example, methods based on deep learning include Stacked Auto-Encoders (SAE)^6^ and Deep Belief Networks (DBN)^7,8^, Convolutional Neural Networks (CNN)^9^ and Random Patches Networks (RPNet)^10^ and so on. This method learns multi-level nonlinear discriminant features unsupervised from HSI data, has better robustness and discriminant, and can improve the classification accuracy of the model. But deep learning methods require a large number of samples, while HSI training samples are limited. If only spectral or spatial features are used for classification, it is prone to the well-known Hughes phenomenon. This paper proposes a new method based on a two-dimensional Gabor filter and random patches convolution (GRPC), which combines spectral-spatial features for HSI image classification.

The specific contributions of the method in this paper are as follows:

1. For the first time, we introduce a GRPC method combining random patches convolution and covariance matrix representation into hyperspectral image classification. GRPC has a simple structure, and the experiments show that its performance can match the state of the art.
2. GRPC not only uses the feature extraction capability of random patches convolution and the advantages of Gabor filters, but also realizes the stacking of spectral and spatial features, revealing the importance of spatial structure features that are usually ignored in HSI classification.
3. GRPC is a competitive and robust method, which can overcome the pepper noise and over-smoothing phenomenon in HSI classification, and it can obtain higher classification accuracy even with limited training samples.

Finally, in order to verify the superiority of GRPC, we also compared with traditional deep learning methods and other classic classification methods. The main ideas for the work to be carried out include the following two aspects: one is to fully consider the benefits of HSI feature information; the other is to improve the classification accuracy of the model while ensuring time efficiency.

The remainder of the paper is organized as follows. In “Related works” section, a comprehensive overview of related work is given. In “Methodology” section, the related technologies, methods and algorithm flow used in this article are described. The information and experimental environment of the data set used are described in “Experimental and analysis” section, which also discusses the experimental parameters and results are discussed. In “Discussion” section, the method of this paper is discussed. Then “Conclusions” section summarizes the paper.

## Related works

HSI classification is a very popular research field in recent years. This section discusses existing methods based on deep learning.

Deep learning algorithms can learn representative and discriminative features hierarchically from data. Through the excellent information representation ability brought by deep structure, the automatic extraction and representation of features can be realized^11^. Designing a reasonable deep network structure can greatly improve the recognition accuracy in classification and target detection applications. For HSI data, the feature extraction of hyperspectral data is extremely difficult due to the high latitude and particularity of hyperspectral images^12^. In order to solve these problems, deep learning algorithms are more and more widely used in HSI feature extraction, classification and target detection. The early deep learning methods used for HSI feature extraction and classification are SAE and DBN. However, the input of SAE and DBN models needs to stretch the three-dimensional structure of the HSI into a one-dimensional feature vector, which will lose the spatial information of the HSI. Different from SAE and DBN input, it is based on the CNN method, which can directly process the input HSI unsupervised and output the classification accuracy^13^. Aiming at the problem of hyperspectral image classification, Hu et al.^14^ first proposed a CNN with a 5-layer network depth to extract the spectral features of HSI, and achieved better classification results when using spectral feature classification. Yue et al.^15^ proposed a method based on 2D-CNN. First, Principal Component Analysis (PCA) was used to reduce the dimensionality of HSI, and the first 3 principal components were retained. The neighborhood extracts spatial features, but the 2D-CNN model alone ignores the spectral information. In order to extract high-order features, Ghasrodashti et al.^16^ constructed a deep autoencoder with multi-layer stacking and spatial update capabilities in an unsupervised manner, and extracted the spectral and spatial by adding similarity angle map criteria, fuzzy patterns and multi-scale feature weights are used to extract spatial and spectral features. Moreover, Roy et al.^17^ proposed a mixed spectral CNN model, using 2D-CNN to extract spectral features and 3D-CNN to extract spatial features, which learns more abstract feature information, and fusing the two types of feature information for HSI classification. This model almost fully utilizes the spectral information and spatial information in the image. However, the CNN model contains a large number of parameters and requires a large number of training samples and training time. Actually, there are fewer training samples in the hyperspectral image, which is very easy to cause over-fitting, resulting in poor generalization of the classification model^18^. Therefore, Ghasrodashti et al.^19^ proposed a sparse classification of hyperspectral images based on extended hidden Markov random fields. The objective function of the sparse classifier was improved by constructing a dictionary containing the minimum spatial-spectral correlation and sparse coding, then satisfactory results were obtained. In order to improve the computational efficiency of the model, Xu et al.^10^ proposed a new RPNet. It uses random projection to determine that the convolution kernel is different from traditional CNN and does not require training. It has excellent feature extraction capabilities to avoid over-fitting problems. And the computational burden of the network is relatively low. It can be seen that this work is flawed. The spatial information in the hyperspectral image is ignored or not used, while the spatial information is the best value information that can be obtained from the hyperspectral image.

With the development of image processing technology, spatial features play an increasingly critical role in HSI classification^20^. Among the existing spatial information extraction technologies, Gabor filters have attracted much attention because of their ability to provide distinctive feature information. Gabor filter is an effective unsupervised feature information extraction method. It has a stronger ability to describe the texture and spatial features, can extract HSI texture and spatial structure information, and reduce the model's limited dependence on training samples^21^. Many researchers have also proved that Gabor filters can achieve better results when applied to HSI classification. For example, Feng Xiao et al.^22^ combined a three-dimensional Gabor filter with a support vector machine (SVM) for hyperspectral image classification, which can effectively improve the classification accuracy and efficiency; Wang Liguo et al.^23^ based on the empirical mode decomposition of the spectral data, the Gabor filtering operation is performed to better mine the texture features of the image; Chen et al.^24^ extracted two-dimensional Gabor features from the hyperspectral data after PCA dimensionality reduction, and input them into 2D-CNN for classification, which improved the classification accuracy and reduced the model’s dependence on training samples and excessive smoothing.

In the field of image processing, it is difficult for a single feature and a single model to achieve the desired performance. An important and effective method is integration^25^. Therefore, in order to solve the above-mentioned problems in the process of hyperspectral image classification, it is inspired by the early fusion of literature^26,27^. This paper proposes a joint spectral-spatial feature method GRPC for HSI classification. This method combines all Gabor spatial features and multi-scale convolution features, and has a high degree of discrimination for HSI classification. In order to avoid the "dimensionality catastrophe" problem^28^, GRPC uses PCA and Linear Discriminant Analysis (LDA) to preprocess the HSI dimensionality reduction, and project the HSI data into a low-dimensional feature space; the Gabor filter is used to extract the texture and internal spatial structure information of the image after dimensionality reduction. At the same time, the Gabor features extracted by the model are used as input, and the deep spectral features of the image are extracted using random patches convolution features. For the extracted spectral features, in order to increase the feature sparsity, a modified linear unit activation function is used for feature activation; finally, the feature stacking method is used to stack spatial features and multi-level convolution features for classification. GRPC not only uses the feature extraction capability of random patches convolution and the advantages of Gabor filters, but also realizes the multi-layer fusion of feature maps, making the network have multi-scale advantages. What needs to be explained here is that the Gabor filter based on frequency domain and direction is similar to the filter in the human visual system. Random patches convolution is similar to CNN and is a network inspired by neuroscience to a large extent. Therefore, the inherent relationship between random patches convolution and Gabor makes the combination of the two technologies have potential advantages over other methods.

## Methodology

### HSI preprocessing

PCA is a common HSI dimensionality reduction method, but often fails to show better category differentiation when dealing with high-dimensional data. The Linear Discriminant Analysis (LDA) algorithm can increase the inter-class to intra-class distance ratio, but the algorithm requires the intra-class distance matrix to be nonsingular, and the data after PCA dimensionality reduction just meets this requirement^29^. Therefore, in this paper, we first apply the PCA algorithm to reduce the dimensionality of the original data and eliminate the redundancy of the data, letting the hyperspectral original input data be ${\text{X}} \in R^{{\left( {rc \times n} \right)}}$, r, c and n, which are the row number, column number and spectral band number respectively, and use the following way to represent the reduced data $X_{P} \in R^{rc \times P}$; then we apply the LDA algorithm to reduce the dimensionality of the data processed by the PCA algorithm using the second projection of $X_{LDA} \in R^{rc \times P}$. This process reduces extra operations and ensures that the intra-class matrix is not singular. The data processed by the two-dimensional reduction process retains the main information and increases the inter-class to intra-class distance ratio, which improves the sample differentiation^30^.

### Gabor filter

The two-dimensional Gabor filter can be used for feature extraction of images, which can effectively capture the asymmetric relationship between the spatial and frequency domains of images and extract intrinsic information such as spatial features, which is an important factor in discriminating different features of HIS^31^. The two-dimensional Gabor filter is composed of an imaginary part and a real part, which is a sinusoidal curve function modulated by a Gaussian envelope, defined as follows.

1 $$G\left( {x,y,\lambda ,\theta ,\psi ,\sigma ,\gamma } \right) = \exp \left( { - \frac{{{\text{x}}^{{{^{\prime}}2}} + {\text{r}}^{2} {\text{y}}^{{{^{\prime}}2}} }}{{2{\upsigma }^{2} }}} \right)\exp \left[ {{\text{i}}\left( {2\prod \frac{{{\text{x}}^{\prime}}}{{\uptheta }} + {\uppsi }} \right)} \right]$$

where ${\text{x}^{\prime}} = {\text{xcos}}\uptheta + {\text{ysin}}\uptheta$ and ${\text{y}^{\prime}} = - {\text{sin}}\uptheta + {\text{ycos}}\uptheta$; ${\uplambda }$ is the wavelength, the wavelength value is specified in pixels, usually greater than or equal to 2, but not greater than one-fifth of the input image size; ${\uptheta }$ is the angle of the Gabor kernel direction, this parameter specifies the direction of the Gabor function parallel stripes, taking a value range of $\left[ {0,{\uppi }} \right]$; ${\uppsi }$ is the phase offset, the default is ${\uppi }/2$; ${\upgamma }$ is the spatial aspect ratio, which determines the ellipticity of the Gabor function shape, when ${\upgamma } = 1$ is, the Gabor kernel shape is circular, when ${\upgamma } < 1$, the shape elongates with the stripe direction, and the default value is 0.5; ${\upsigma }$ is the standard deviation of the Gaussian envelope, determined by the wavelength ${\uplambda }$ and the spatial frequency bandwidth ${\text{b}}_{{\text{w}}}$, defined as follows.

2 $${\upsigma } = \frac{{\uplambda }}{{\uppi }}\sqrt {\frac{{{\text{ln}}2}}{2}} \cdot \frac{{2^{{{\text{b}}_{{\text{w}}} }} + 1}}{{2^{{{\text{b}}_{{\text{w}}} }} - 1}}$$

where the value of the bandwidth ${\text{b}}_{{\text{w}}}$ is a positive real number, the smaller the bandwidth, the larger the standard deviation, and the larger the shape of the Gabor kernel; when its value is taken in $\left[ {1,5} \right]$, it does not affect the results of the algorithm in this paper much, and its default value is 1.

### Random patches convolution

Unlike traditional convolutional feature extraction, the convolutional kernel of the random patches convolution part is determined using random projection, the basic principle of which is derived from the JL Lemma and is an effective method for dimensionality reduction^32^. Arriaga et al.^33^ achieved classification by projecting data into a random low-dimensional space. It is proved that the random projection can well preserve the boundary between different categories, and only a small number of samples are needed to train the classifier in the low-dimensional space. This feature is conducive to image classification tasks, especially in the case of very limited training samples. The random patches convolution part used in this paper mainly consists of data PCA whitening, random projection and convolutional feature extraction, as described below.

PCA data whitening: PCA whitening refers to the standardization of the features of each dimension after PCA dimensionality reduction. In this way, the variances of different wavebands are similar, and the correlation between different wavebands is reduced, which is conducive to the completion of the image classification task.

Random projection and convolutional feature extraction: The random patches used in this paper are randomly selected from the PCA-whitened data ${\text{X}}_{{{\text{whiten}}}}$ using a random function with k random pixels ${\text{X}}_{{{\text{whiten}}}} \in {\text{R}}^{{{\text{rc}} \times {\text{P}}}}$, and around each pixel, a ${\text{w}} \times {\text{w}} \times {\text{P}}$ patches is taken to obtain k random patches ${\text{P}}_{1} ,{\text{P}}_{2} , \ldots ,{\text{P}}_{{\text{k}}} \in {\text{R}}^{{{\text{w}} \times {\text{w}} \times {\text{P}}}}$. For those pixels distributed at the edge of the image, a mirror image is used to fill in the neighboring blank pixels. Then, all k random patches are used as convolution kernels to convolve the whitened data with the random patches to obtain k feature maps

3 $${\text{I}}_{{\text{i}}} = \mathop \sum \limits_{{{\text{j}} = 1}}^{{\text{P}}} {\text{X}}_{{{\text{whiten}}}}^{{\text{j}}} {\text{*P}}_{{\text{i}}}^{{\left( {\text{j}} \right)}} ,{\text{i}} = 1,2, \cdots \cdots ,{\text{k}}$$

${\text{I}}_{{\text{i}}} \in {\text{R}}^{{\left( {{\text{r}} \times {\text{c}}} \right)}}$ is the i-dimensional feature map, ${\text{X}}_{{{\text{whiten}}}}^{{\text{j}}} \in {\text{R}}^{{{\text{r}} \times {\text{c}}}}$ is the jth dimension of the downscaled data, and ${\text{P}}_{{\text{i}}}^{{\left( {\text{j}} \right)}} \in {\text{R}}^{{{\text{w}} \times {\text{w}}}}$ is the jth dimension of the i-th random patches. The step size of the convolution operation is set to 1. For blank pixels at the edges, they are filled by mirroring the image.

### Hyperspectral images classification method based on GRPC

**Figure 1:**
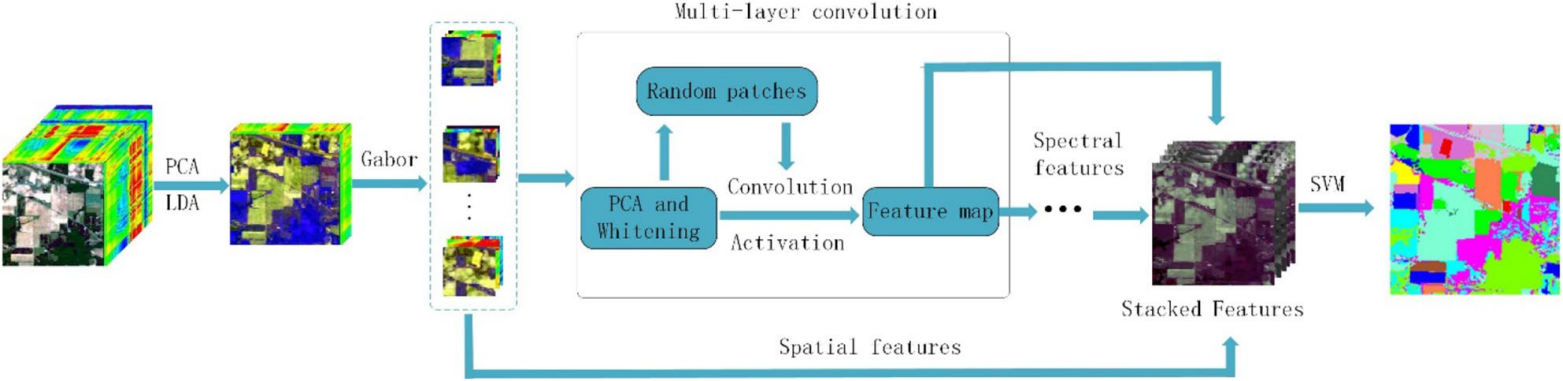
The method flow chart of this paper.

In order to make full use of the effectiveness of HSI spectral and spatial features and improve the classification accuracy of HSI, this paper combines a two-dimensional Gabor filter with random patches convolution feature extraction, and proposes a new joint spatial and spectral feature information HSI classification method GRPC. The steps of this method are shown in Fig. 1, which mainly includes four steps: First, use PCA and LDA algorithms to reduce the dimensionality of the input original HSI, and retain the main information of the HSI, eliminating the redundancy of the data, and increase the inter-class and the distance ratio within the class; secondly, the two-dimensional Gabor filter is used to extract the spatial structure feature information of the image after the dimensionality is reduced; again, the extracted Gabor features are used as input, and the multi-level spectral feature information of the image is extracted using random patches convolution features; finally, connect spatial and spectral features to perform feature stacking, then use SVM classifier to predict category labels, and output classification accuracy.

Among them, the random patches convolution feature extraction part contains multiple layers, and each layer contains a PCA, whitening, random projection and convolution feature extraction. The method of this paper uses a modified linear unit activation for the convolved feature map. The function performs feature activation, the activation function can be expressed as $f\left( I \right) = \max \left( {0,I - S} \right)$, ${\text{S}} = \left[ {S_{2} \cdots S_{2} } \right]$ is an average matrix composed of k $S_{2}$, and $S_{2}$ is the mean vector of I in the second dimension, K is the number of feature maps, if I is the convolution feature of the first layer, then $Z_{1} = f\left( I \right)$. Feature stacking can better meet the precise classification requirements of different targets at different scales by combining spectral and spatial features in classification^34^. The feature stacking of GRPC is to transform the acquired spectral and spatial feature information and the original spectral data into the same dimension for splicing. If Z is regarded as the final feature vector used in classification, the final feature vector in each pixel $\left( {{\text{i}},{\text{j}}} \right)$ is as follows, ${\text{M}}_{{\left( {{\text{i}},{\text{j}}} \right)}} = \left\{ {{\text{G}}_{1} , \ldots ,{\text{G}}_{{\text{R}}} ,{\text{Z}}_{1} , \ldots ,{\text{Z}}_{{\text{L}}} ,{\text{X}}} \right\}$; ${\text{G}}_{{\text{R}}}$ are different Gabor features, ${\text{Z}}_{{\text{L}}}$ is the convolution feature of different layers, and L is the number of network layers extracted by the convolution feature. It is worth noting that these features still need to be standardized, and the standardized formula is shown in formula (4).

4 $${\text{M}}_{{\left( {{\text{i}},{\text{j}}} \right)}}^{{{\text{norm}}}} = \frac{{{\text{M}}_{{\left( {{\text{i}},{\text{j}}} \right)}} - {\text{mean}}\left( {{\text{M}}_{{\left( {{\text{i}},{\text{j}}} \right)}} } \right)}}{{{\text{var}}\left( {{\text{M}}_{{\left( {{\text{i}},{\text{j}}} \right)}} } \right)}}$$

${\text{M}}_{{\left( {{\text{i}},{\text{j}}} \right)}}^{{{\text{norm}}}}$, ${\text{var}}\left( {{\text{M}}_{{\left( {{\text{i}},{\text{j}}} \right)}} } \right)$ and ${\text{mean}}\left( {{\text{M}}_{{\left( {{\text{i}},{\text{j}}} \right)}} } \right)$ are the standardized, ${\text{M}}_{{\left( {{\text{i}},{\text{j}}} \right)}}$ variance and mean, respectively. Finally, the standardized final features are used to classify the HSI using the SVM classifier. The SVM classifier has the advantages of simple calculation and good classification performance. The implementation details of the GRPC method are shown in Algorithm 1.


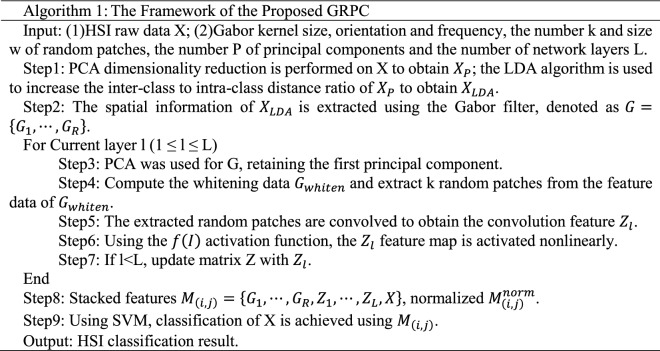


## Experimental and analysis

### Data and experimental environment description

**Figure 2:**
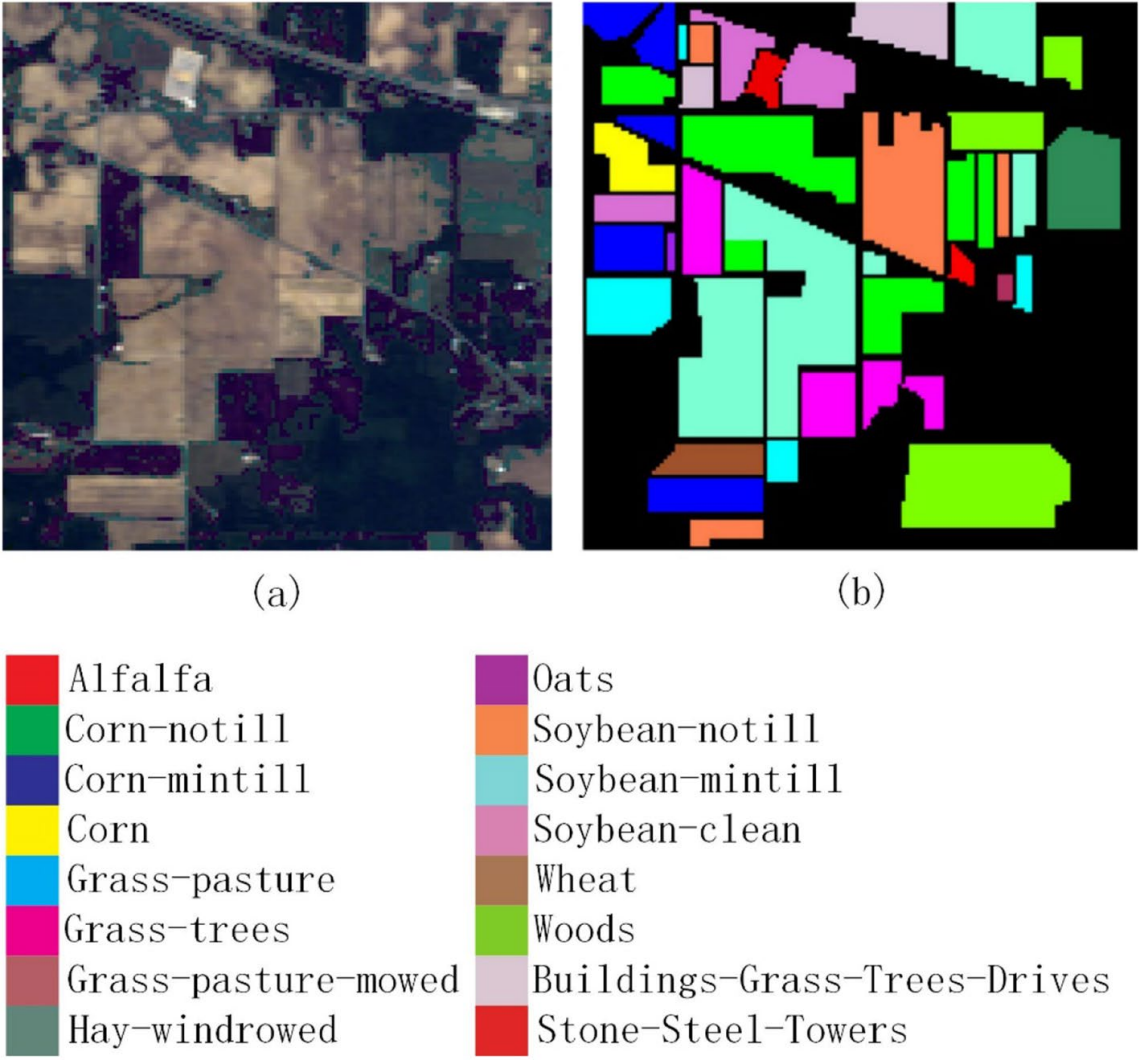
Indian Pines data set. (**a**) False-color map; (**b**) Ground-truth map.

**Figure 3:**
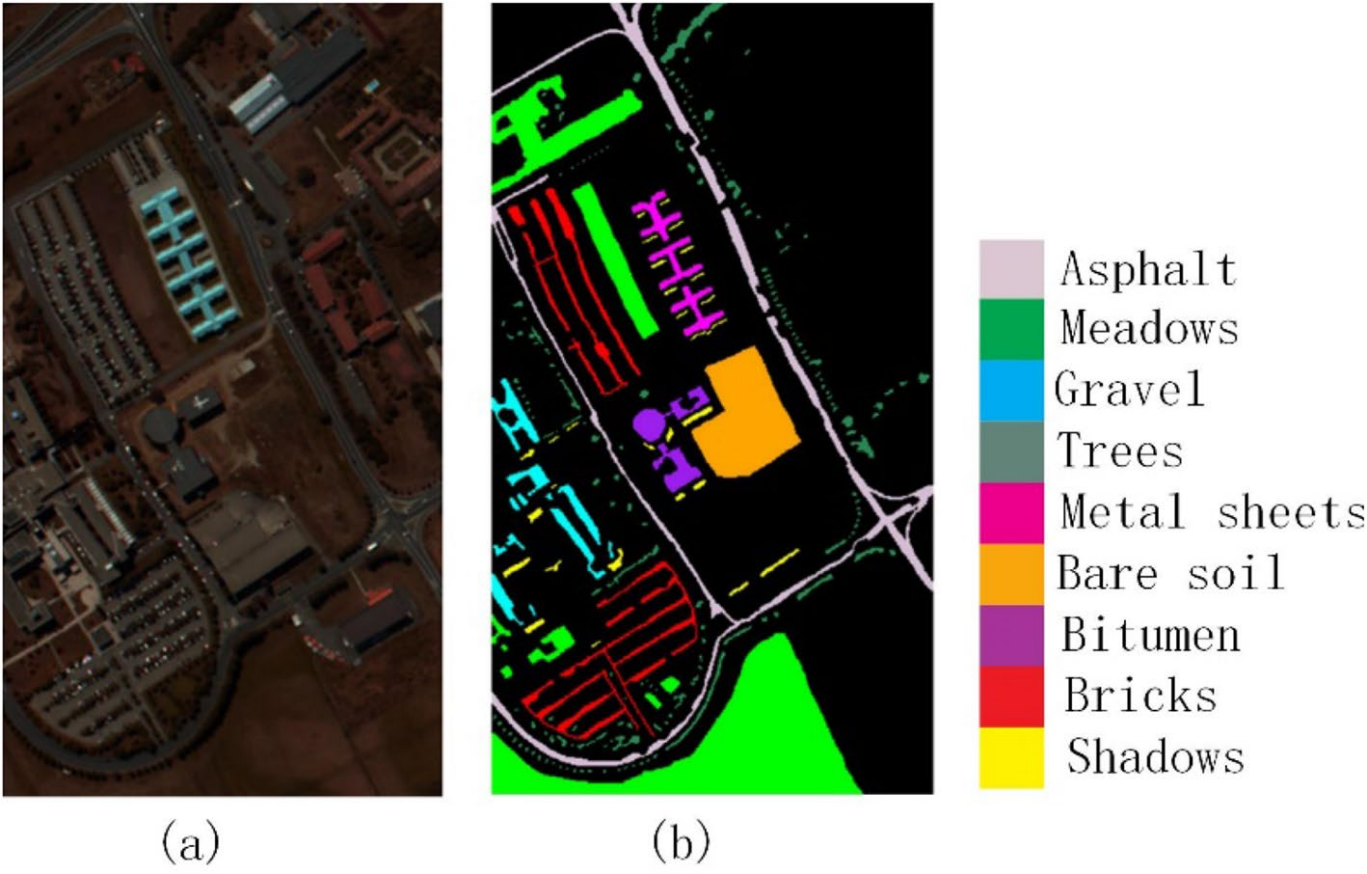
Pavia University data set. (**a**) False-color map; (**b**) Ground-truth map.

**Figure 4:**
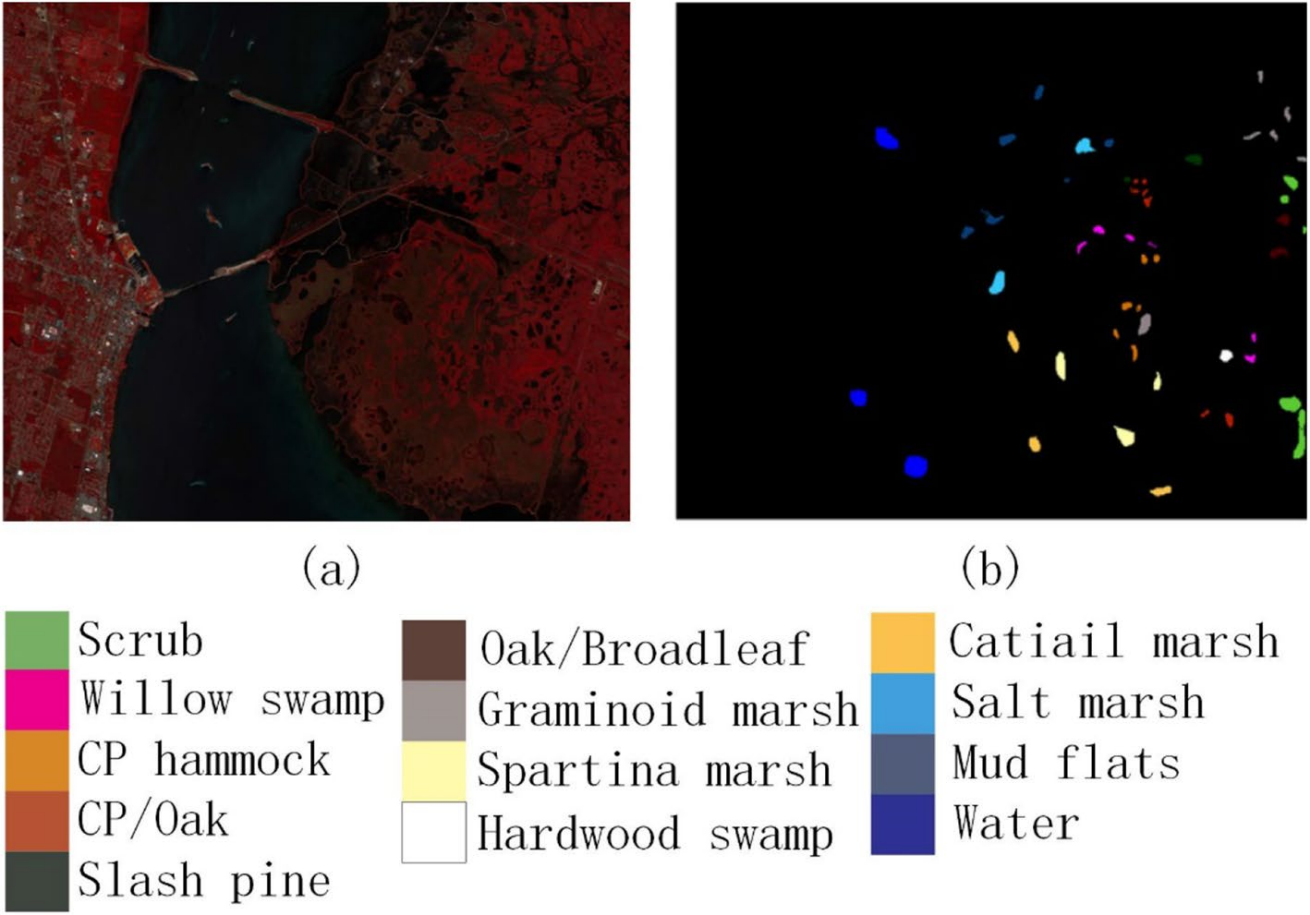
Kennedy Space Center. (**a**) False-color map; (**b**) Ground-truth map.

In order to verify the effectiveness of the method proposed in this paper, experiments were conducted on the three most widely used public data sets of Indian Pines^35^, Pavia University^35^ and Kennedy Space Center^35^. The three data sets are all images with the absorption band removed from water mist. The Indian Pines image was obtained from an AVIRIS spectrometer in a forest area in northwestern Indiana, USA. The image size is 145 × 145 pixels, the wavelength is 0.40–2.50 μm, and the number of bands is 200, the spatial resolution is 20 m; the Pavia University image is obtained by ROSIS spectrometer at the University of Pavia, Italy. The image size is 610 × 340 pixels, the wavelength is 0.43–0.86 μm, the number of bands is 103, and the spatial resolution is 1.3 m; the Kennedy Space Center image was obtained by an AVIRIS spectrometer near the Kennedy Space Center in Florida. The image size is 512 × 614 pixels, the wavelength is 0.40–2.50 μm, the number of bands is 176, and the spatial resolution is 18.0 m. The three data sets are divided into 16 categories, 9 categories and 13 categories. Their false-color images and ground-truth images are shown in Figs. 2, 3, and 4, respectively. The numbers of training samples and test samples are shown in Tables 1, 2 and 3.

**Table 1:** Number of training and testing samples in the Indian Pines Dataset.

Class number	Class name	Training	Test
1	Alfalfa	30	16
2	Corn-notill	150	1278
3	Corn-mintill	150	680
4	Corn	100	137
5	Grass-pasture	150	333
6	Grass-trees	150	580
7	Grass-pasture-mowed	20	8
8	Hay-windrowed	150	328
9	Oats	15	5
10	Soybean-notill	150	822
11	Soybean-mintill	150	2305
12	Soybean-clean	150	443
13	Wheat	150	55
14	Woods	150	1115
15	Buildings-Grass-Trees-Drives	50	336
16	Stone–Steel-Towers	50	43
	Total	1765	8484

**Table 2:** Number of training and testing samples in the Pavia University Dataset.

Class number	Class name	Training	Test
1	Asphalt	548	6083
2	Meadows	540	18,109
3	Gravel	392	1707
4	Trees	542	2522
5	Metal sheets	256	1089
6	Bare soil	532	4497
7	Bitumen	375	955
8	Bricks	514	3168
9	Shadows	231	716
	Total	3930	38,846

**Table 3:** Number of training and testing samples in the Kennedy Space Center Dataset.

Class number	Class name	Training	Test
1	Scrub	33	728
2	Willow swamp	23	220
3	CP hammock	24	232
4	CP/Oak	24	228
5	Slash pine	15	146
6	Oak/Broadleaf	22	207
7	Hardwood swamp	9	96
8	Graminoid marsh	39	393
9	Spartina marsh	51	469
10	Catiail marsh	39	365
11	Salt marsh	41	378
12	Mud flats	49	454
13	Water	91	836
	Total	459	4752

Regarding the False-color map and Ground-truth map in Figs. 2, 3, 4, 8, 9 and 10, we used Python to draw them ourselves. The software version is Python 3.7.6^36^. The website link is https://www.python.org/downloads/release/python-376/.

The whole experiment process was carried out on a local PC. The operating system was Windows10 Enterprise Edition, and the hardware configuration was Intel Core i5-4570S @ 2.90 GHz, NVIDIA GeForce GT 750 M and 8 GB RAM. Code will be available at https://github.com/Shenming-Qu/GRPC.

### Parameter analysis

**Figure 5:**
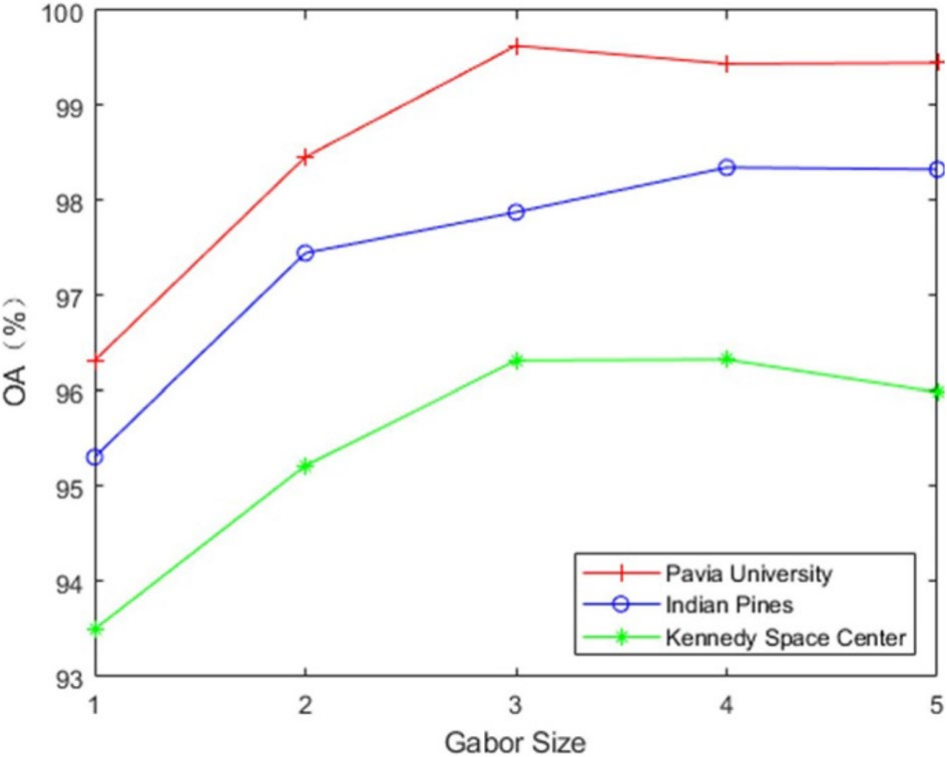
The effect of Gabor window size on three data sets on classification accuracy.

Gabor Filter: In general, a Gabor filter is considered to be a multi-directional and multi-scale filter^20^. In this paper, in order to reduce the computational complexity of the network while maintaining a robust feature representation capability, a multi-directional and single-scale Gabor filter is used. By testing a combination of three center frequencies (0.1, 0.2 and 0.4 Hz) and two sets of filter directions (four directions θ = 0, π/4, π/2, 3π/4 and eight directions θ = 0, π/8, π/4, 3π/8, π/2, 5π/8, 3π/4, 7π/8), this article uses a filter bank of 0.2 Hz and four directions. In addition, the influence of Gabor window size on classification accuracy is evaluated. As shown in Fig. 5, with the window size changes, the classification accuracy of Indian Pines and Pavia University increases first and then stabilizes. The classification accuracy of Kennedy Space Center has increased after it has decreased. The reason is that if the window is too small, it will highlight the details in the image, but it is susceptible to noise interference. If the window is too large, it is not conducive to express the local characteristics of the image and lose the details of the image. Finally, the window size is set to 3 × 3.

**Figure 6:**
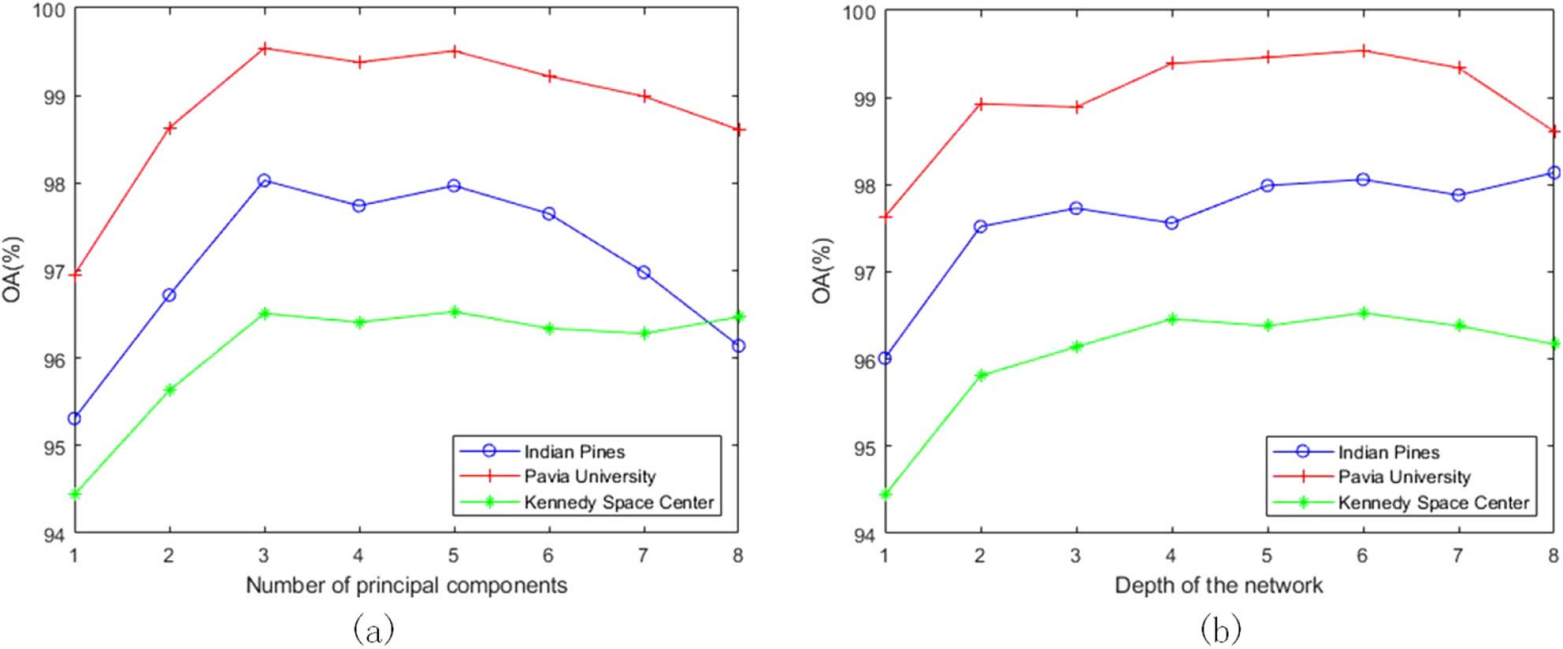
Effect of number of principal components and number of network layers on classification accuracy on three datasets. (**a**) Number of principal components; (**b**) Network layers.

Number of PCA Principal Components and Number of Network Layers: This paper analyzes the influence of the principal component parameters and the number of network layers L on the classification accuracy through experiments. As shown in (a) of Fig. 6, with the gradual increase of the parameter P, the OA of this method in Kennedy Space Center gradually stabilizes, and the OA of the two data sets of Indian Pines and Pavia University has a significant downward trend. As the value of P increases, the experimental time of the three data sets also becomes significantly longer. Considering the balance between accuracy and time consumption, this article sets P to 3. As shown in Fig. 6b, the classification accuracy increases as the number of network layers increases. When the number of network layers is less than 6, the classification accuracy gradually increases and tends to be stable. When the number of network layers is greater than 6, Pavia University and Kennedy Space Center drop significantly. This shows that although too deep network layers can extract more abstract feature information, it will also cause loss of information, which will affect the classification accuracy. Finally, the parameter L is set to 6.

**Figure 7:**
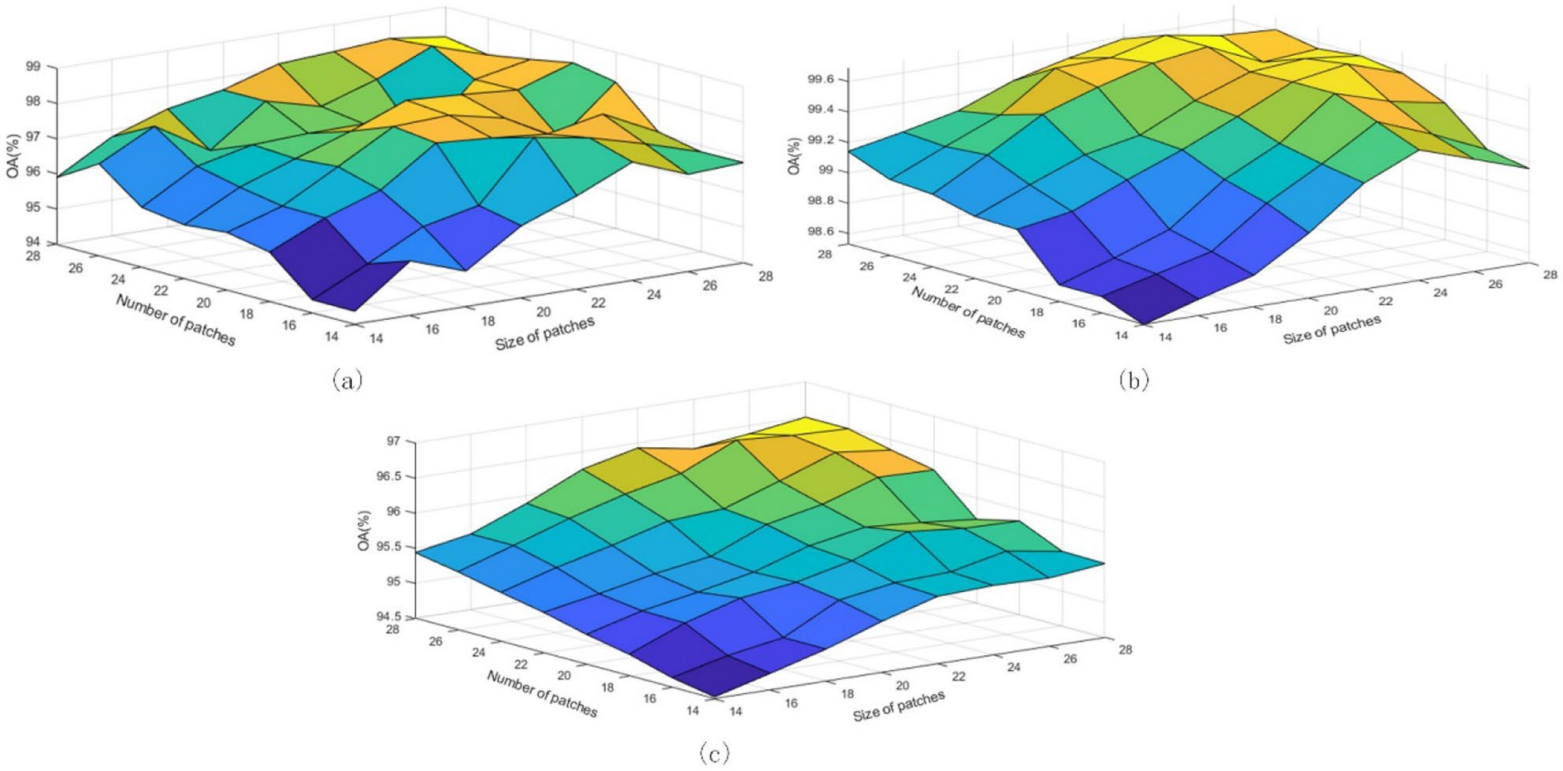
The effect of random patches size and number on classification accuracy on three data sets. (**a**) Indian Pines; (**b**) Pavia University; (**c**) Kennedy Space Center.

Number and Size of Random Patches: The number k and size w of random patches are two important parameters of GRPC, and the size of these two parameters will affect the classification accuracy. In this paper, we experimentally evaluate the impact of different k and w on the method in this paper. As shown in Fig. 7, a smaller k cannot achieve higher classification accuracy, and in this experiment, the parameter k is set to 23. As for the parameter w, in general, a larger w can help to improve the classification accuracy, but too large a w can increase the phenomenon of transition smoothing, and in this experiment, the parameter w is set to 24.

### Evaluation metrics

In order to better evaluate the performance of this method in the experiment, two indicators, Overall Accuracy (OA) and Kappa Coefficient (Kappa) are used to evaluate the classification performance of the model.

OA is equal to the sum of correctly classified pixels divided by the total number of pixels. The number of correctly classified pixels is distributed along the diagonal of the confusion matrix, and the total number of pixels is equal to the total number of pixels of all real reference sources. The formula is as follows, where h_ii_ is the number of correctly classified pixels distributed along the diagonal of the confusion matrix, N is the total number of samples, and n is the number of categories.

5 $${\text{OA}} = \frac{{\mathop \sum \nolimits_{j = 1}^{n} h_{ii} }}{N} \times 100{\text{\% }}$$

Kappa coefficient is an indicator of consistency test. It is done by multiplying the total number (N) of all real reference pixels by the sum of the diagonal (h_kk_) of the confusion matrix, and then subtracting the number of real reference pixels in each category and the classified pixels (h_ik_, h_kj_) after the product of the total number, divided by the square of the total number of pixels minus the product of the total number of true reference pixels in each category and the total number of classified pixels in the category, and then sum the result. The kappa coefficient comprehensively considers the various factors in the confusion matrix and can more comprehensively reflect the accuracy of the overall classification. The larger the value of the Kappa coefficient, the higher the accuracy of the corresponding classification algorithm. The general formula is as follows.

6 $${\text{Kappa}} = \frac{{N\mathop \sum \nolimits_{k} h_{kk} - \mathop \sum \nolimits_{k} \left( {\mathop \sum \nolimits_{j} h_{kj} \mathop \sum \nolimits_{i} h_{ik} } \right)}}{{N^{2} - \mathop \sum \nolimits_{k} \left( {\mathop \sum \nolimits_{j} h_{kj} \mathop \sum \nolimits_{i} h_{ik} } \right)}}$$

### Classification results

In order to verify the advantages of this method in the classification of hyperspectral remote sensing images, this method is compared with the current mainstream PCA (using RBF-SVM to classify the first 20 principal components), EMP^37^, SAE^38^, 3D-CNN^39^, Gabor-Based^21^ and RPNet^10^ and other methods are compared. Among them, PCA and EMP are two classic and representative machine learning methods; SAE is a classic machine learning method; 3D-CNN achieves HSI classification only by extracting local spectral-spatial information of pixels; Gabor-Based has the same data input as the method in this paper; RPNet uses spectral feature information for classification, and has the same model structure as the convolution feature extraction module in the method in this paper, but the difference lies in the input data. The number of training samples and the number of test samples for each method remain the same. The number of detailed training samples and test samples is shown in Tables 1, 2 and 3. The classification accuracy of different methods on the three data sets is shown in Tables 4, 5 and 6. The bold font in the table represents the highest value in the same industry. The following comparative analysis can draw conclusions.

**Table 4:** Classification accuracy of Indian Pines Dataset.

Class	PCA	EMP^37^	SAE^38^	3D-CNN^39^	Gabor-Based^21^	RPNet^10^	Ours
1	83.54	**100**	**100**	93.75	87.50	96.25	**100**
2	67.79	91.63	82.86	93.97	94.60	94.02	**96.40**
3	72.74	94.41	94.10	90.74	97.79	96.59	**98.82**
4	80.87	97.08	92.63	97.08	97.08	99.20	**100**
5	93.88	98.80	97.15	98.80	98.80	99.00	**99.10**
6	96.02	**100**	98.60	99.48	**100**	99.69	98.79
7	87.91	87.50	98.75	87.50	87.50	91.67	**100**
8	97.90	**100**	99.79	**100**	**100**	99.91	**100**
9	80.66	**100**	**100**	**100**	**100**	**100**	**100**
10	73.23	94.28	90.29	95.26	95.99	96.36	**98.78**
11	62.04	91.67	84.39	91.67	91.80	93.08	**96.83**
12	74.45	97.52	93.93	97.29	98.42	97.72	**98.87**
13	98.30	98.18	99.64	**100**	**100**	99.70	**100**
14	93.40	98.39	96.78	98.92	99.10	99.21	**99.91**
15	46.54	94.05	85.51	83.93	93.15	95.76	**97.62**
16	91.39	**100**	99.77	**100**	97.67	98.37	97.67
OA/%	74.72 ± 0.99	94.78 ± 0.62	90.11 ± 0.24	94.56 ± 0.34	95.78 ± 0.29	96.09 ± 0.66	**98.09 ± 0.32**
Kappa × 100	71.05 ± 1.18	93.95 ± 0.83	88.56 ± 0.28	93.68 ± 0.4	95.11 ± 0.37	95.46 ± 0.76	**97.78 ± 0.37**

**Table 5:** Classification accuracy of Pavia University Dataset.

Class	PCA	EMP	SAE	3D-CNN	Gabor-Based	RPNet	Ours
1	87.29	98.11	92.25	97.80	97.04	98.35	**99.49**
2	90.77	98.43	91.19	97.34	98.98	99.52	**99.59**
3	75.37	97.94	89.39	96.66	97.72	98.97	**99.65**
4	97.43	99.53	98.87	99.05	**99.68**	99.41	99.52
5	99.87	**100**	99.97	**100**	**100**	99.99	99.91
6	91.52	99.34	93.28	97.87	99.56	99.88	**100**
7	87.01	99.40	96.39	**99.90**	99.27	99.47	**99.90**
8	79.28	98.02	96.39	97.92	97.95	99.20	**99.59**
9	99.88	99.87	99.80	98.74	99.86	99.90	**100**
OA/%	89.46 ± 0.36	98.59 ± 0.27	92.97 ± 0.93	97.76 ± 0.29	98.70 ± 0.25	99.34 ± 0.12	**99.64 ± 0.14**
Kappa × 100	85.82 ± 0.46	98.10 ± 0.33	90.55 ± 1.19	96.96 ± 0.37	98.22 ± 0.33	99.10 ± 0.17	**99.51 ± 0.20**

**Table 6:** Classification accuracy of Kennedy Space Center Dataset.

Class	PCA	EMP	SAE	3D-CNN	Gabor-Based	RPNet	Ours
1	89.97	92.72	92.58	89.84	91.62	89.70	**95.08**
2	91.36	83.18	90.00	**95.00**	86.82	85.45	94.30
3	85.34	84.05	86.21	92.24	97.41	94.83	**97.57**
4	79.82	87.72	78.07	81.14	84.65	85.09	**94.55**
5	69.86	80.82	82.88	95.21	77.40	**99.32**	98.20
6	59.42	73.91	57.49	61.84	70.05	78.74	**86.03**
7	75.00	71.88	78.13	71.88	**87.50**	78.13	87.36
8	92.11	90.59	88.55	92.11	91.09	92.62	**94.75**
9	97.01	97.44	98.93	**99.79**	94.67	98.08	95.10
10	81.37	94.25	93.97	98.08	96.16	95.89	**99.15**
11	96.83	98.41	95.50	98.94	94.18	98.68	**100**
12	88.99	96.26	90.09	96.48	95.81	**100**	97.57
13	99.64	99.52	99.52	99.76	99.52	**99.88**	**99.88**
OA/%	89.44 ± 0.64	92.40 ± 0.44	90.95 ± 0.36	93.26 ± 0.29	92.49 ± 0.71	94.15 ± 0.84	**96.53 ± 0.75**
Kappa × 100	88.24 ± 0.72	91.64 ± 0.56	89.92 ± 0.46	92.50 ± 0.37	91.63 ± 0.81	93.49 ± 0.93	**96.12 ± 0.87**

The OA and Kappa coefficients of the method in this paper are significantly higher than those of the compared methods. On the Indian Pines dataset, the method achieved an OA of 98.09% and a Kappa coefficient of 0.9778, an improvement of 2% and 0.0232 compared to RPNet, and 2.31% and 0.0267 compared to Gabor-Based; for the Pavia University dataset, the OA reached 99.64% and the Kappa coefficient reached 0.9951, an improvement of 0.30% and 0.0041 compared to RPNet, and 0.94% and 0.0129 compared to Gabor-Based; for the Kennedy Space Center dataset, OA reached 96.53% and the Kappa coefficient reached 0.9612, an improvement of 2.38% and 0.38% compared to RPNet improved by 2.38% and 0.0263, compared to Gabor-Based by 3.86% and 0.0449, etc.

The Gabor filter and random patches convolution included in the model in this paper have good feature extraction capabilities for HSI. Moreover, the method in this paper achieves higher classification accuracy than RPNet and Gabor-Based, and also demonstrates the advantage of using the combination of Gabor filters and RPNet to extract spatial information including edges and textures.

This method is also able to improve the classification accuracy of a variety of features, such as Alfalfa, Grass-pasture-mowed and Soybean-mintill in the Indian Pines dataset; Asphalt, Gravel and Bitumen in the Pavia University dataset; and Scrub, CP/Oak and Salt marsh in the Kennedy Space Center dataset. Features such as Scrub, CP/Oak and Salt marsh in the Kennedy Space Center dataset. It also demonstrates the advantage of using multi-level features to bring different feature information at different scales to the classification accuracy.

**Figure 8:**
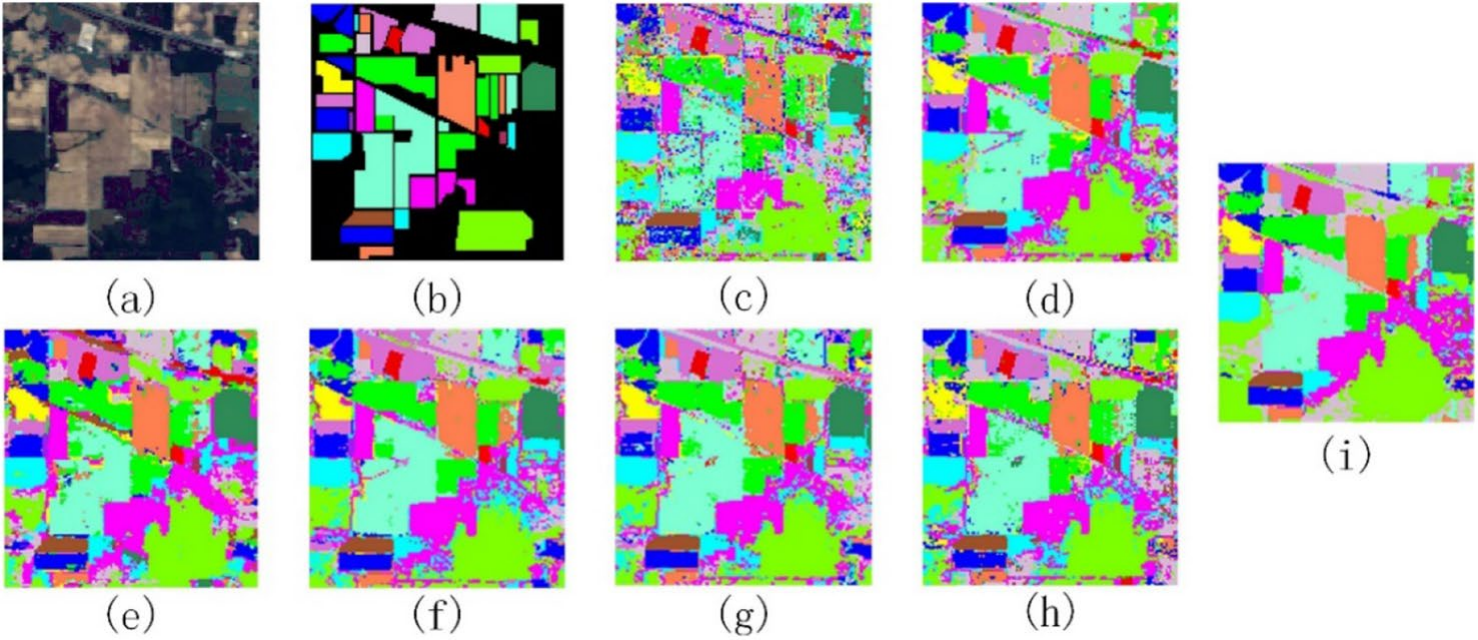
Indian Pines data classification results. (**a**) False-color map; (**b**) Ground-truth map; (**c**) PCA; (**d**) EMP; (**e**) SAE; (**f**) 3D-CNN; (**g**) Gabor-Based; (**h**) RPNet; (**i**) Ours.

**Figure 9:**
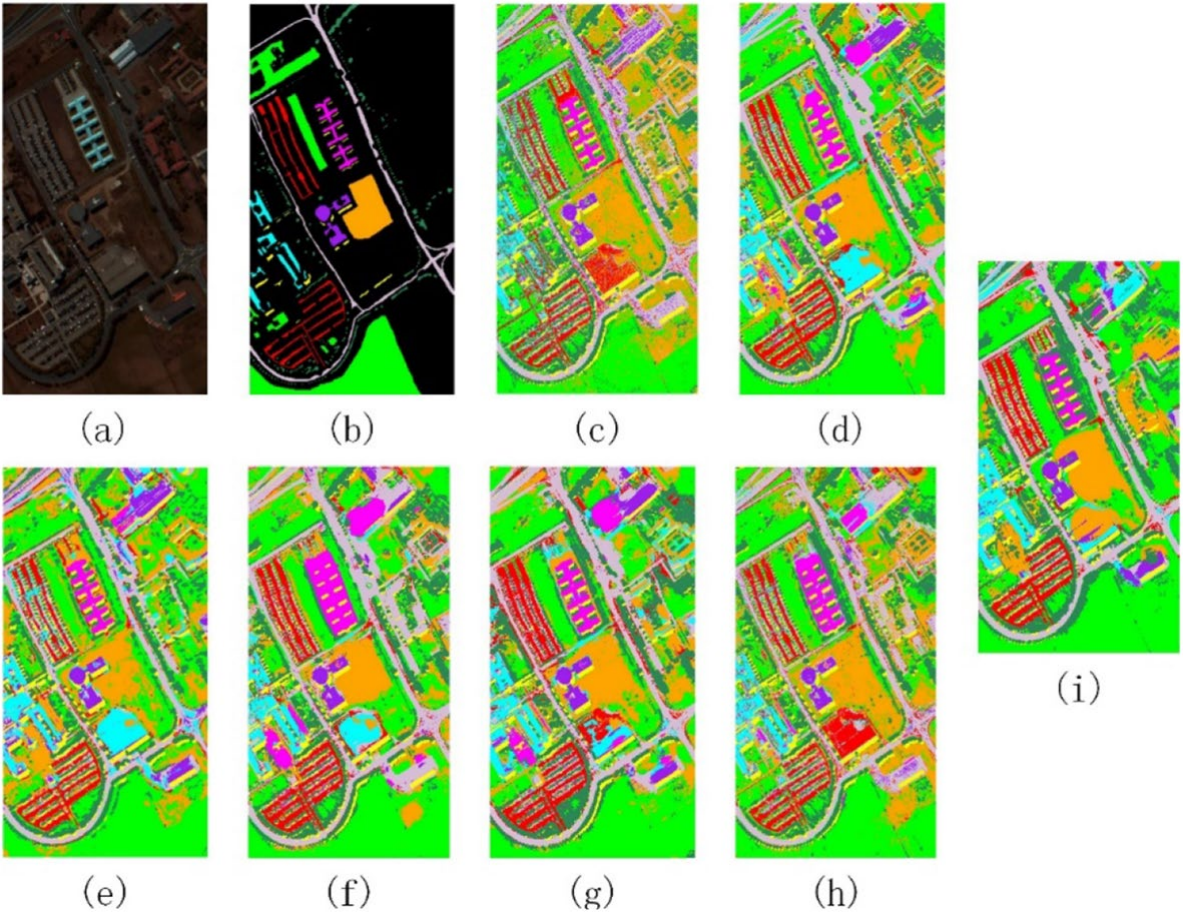
Pavia University data classification results. (**a**) False-color map; (**b**) Ground-truth map; (**c**) PCA; (**d**) EMP; (**e**) SAE; (**f**) 3D-CNN; (**g**) Gabor-Based; (**h**) RPNet; (**i**) Ours.

**Figure 10:**
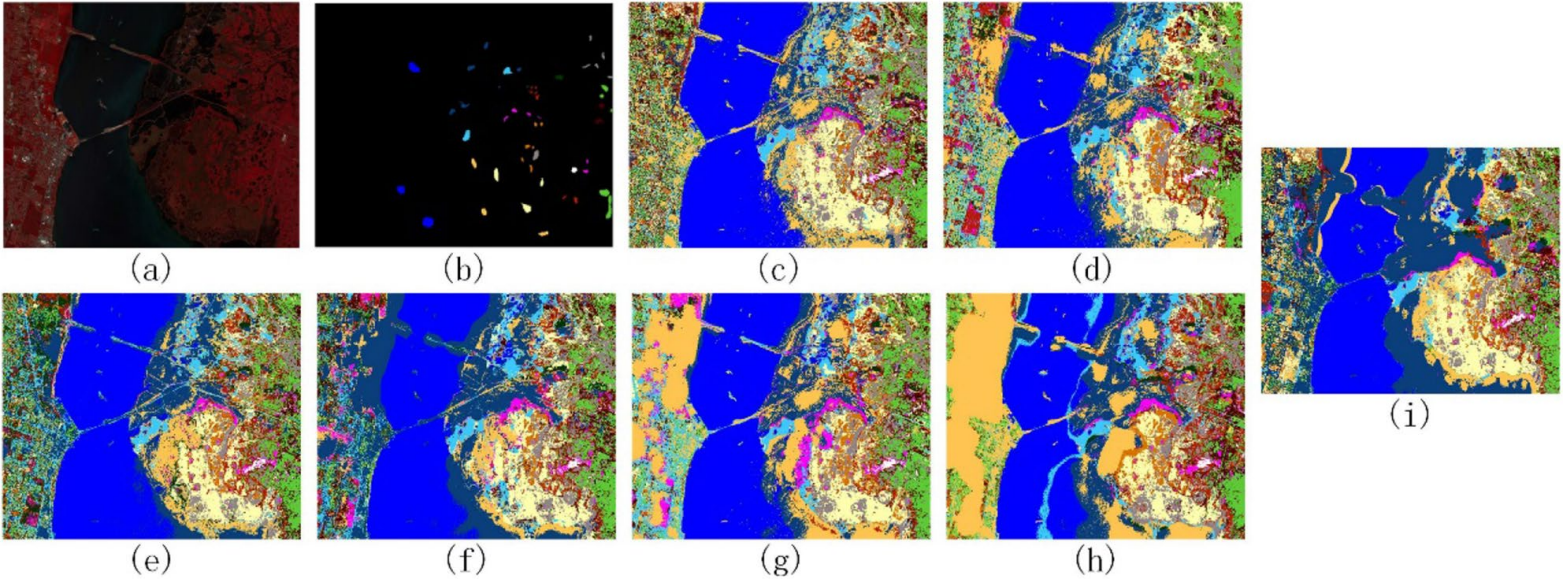
Kennedy Space Center data classification results. (**a**) False-color map; (**b**) Ground-truth map; (**c**) PCA; (**d**) EMP; (**e**) SAE; (**f**) 3D-CNN; (**g**) Gabor-Based; (**h**) RPNet; (**i**) Ours.

Figures 8, 9 and 10 show the ground truth sample plots of the three data sets and the classification result plots obtained by comparing the methods and the method of this paper respectively. From the classification result plots in Figs. 8, 9 and 10, it can be seen that the method in this paper does not have large blocks of noise points in the classification result plots, and the classification results of all types of features have high classification accuracy, which is consistent with the classification accuracy results in Tables 4, 5 and 6.

**Figure 11:**
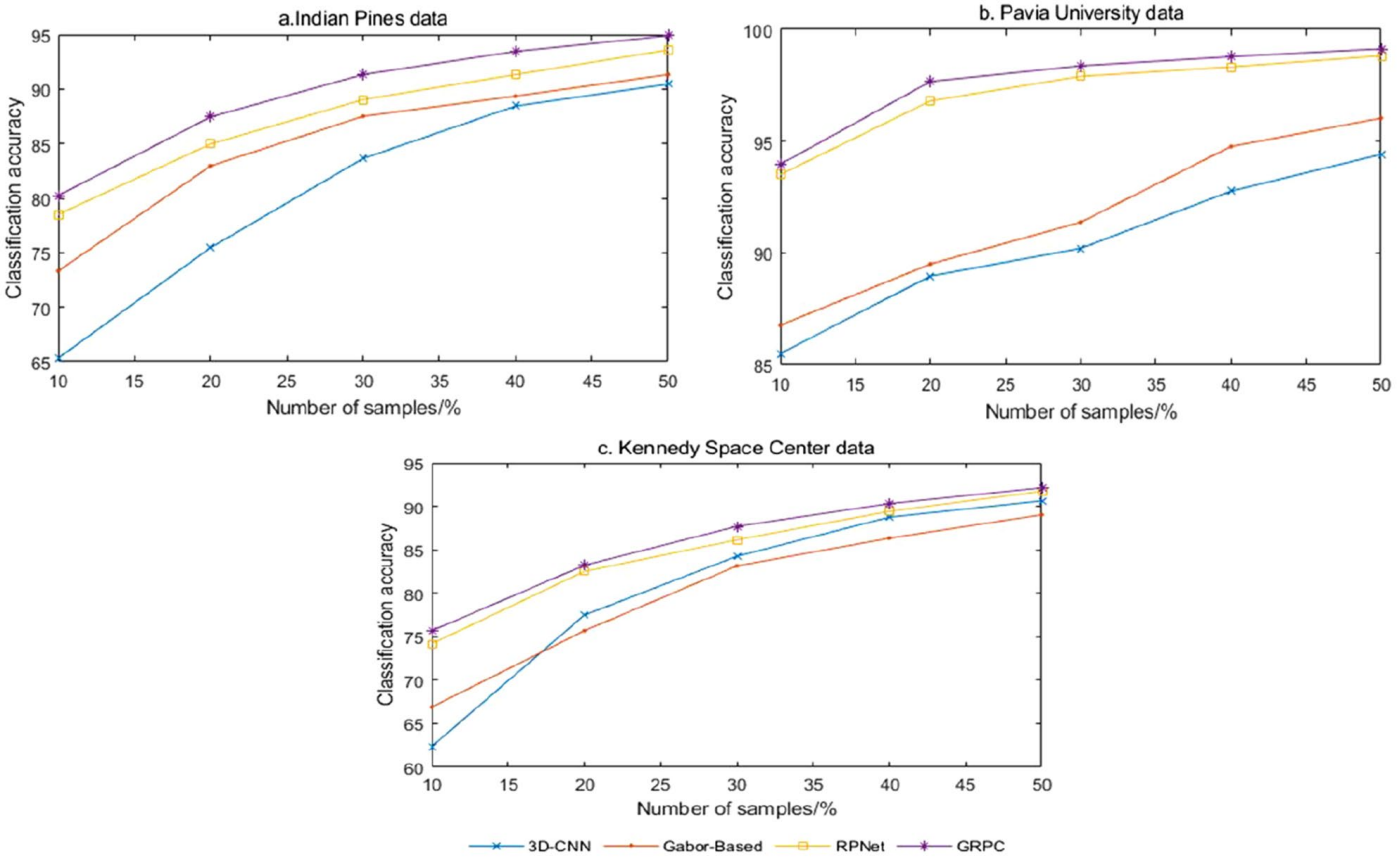
Accuracy with different number of training samples.

Due to the high cost of acquiring hyperspectral datasets, the number of samples available is very limited. In order to further investigate the robustness of the algorithm of this paper's method under different numbers of training samples, another 10%, 20%, 30%, 40% and 50% of the training samples of each type of feature were selected as training samples on three datasets of Indian Pines, Pavia University and Kennedy Space Center, respectively, and compared with 3D-CNN, Gabor-Based and RPNet methods for comparison experiments. Ten experiments were conducted for each method with different training sample ratios, and the average of OA accuracy was taken after the experiments for comparison. Figure 11 shows the accuracy rates under different training samples. It can be seen from Fig. 11 that the OA accuracy rises gradually as the number of training samples increases, and the method in this paper achieves higher classification accuracy with different numbers of training samples and has obvious advantages. It can be seen that this method has better robustness under the limited number of samples.

## Discussion

In Figs. 7, 8, 9, 10, 11 and Tables 4, 5, 6, the comparison of GRPC in the three data sets with several other comparison methods shows that the GRPC method can achieve better visual effects and higher accuracy. This proves the effectiveness of the spectral-spatial feature extraction mode in the GRPC method. There are three reasons for this. First, the two-dimensional Gabor filter is used to extract the spectral-spatial feature information of the image, so as to obtain the local spatial correlation information of all regions of the entire image. Second, random patches convolution can extract shallow and deep features, allowing the combination of multi-scale and multi-layer features. Third, random patches convolution and Gabor localized spatial features in GRPC have great potential for overcoming the pepper noise and over-smoothing phenomenon in HSI processing, and a good classification effect is still achieved with limited training samples. Through the above classification results and quantitative evaluation, the method in this paper can become a novel and effective spectral-spatial classification framework.

In order to verify the superiority of GRPC spatial-spectral joint feature classification and the influence of LDA during data preprocessing. Three different methods were also designed as variants of GRPC in the experiment. One is that NG-RPC that does not use Gabor features in the feature stacking part, and the parameter settings are the same as GRPC. Second, OGRPC, which only stacks Gabor features and random patches convolution deep features, is different from GRPC in that only the last layer of features of random patches convolution is used in the feature stacking part. Third, in the data preprocessing part, only PCA dimensionality reduction is used to preprocess the input image PGRPC. Table 7 shows the classification accuracy of the four methods. It can be seen from Table 7. The two simple feature fusion methods, NG-RPC and OGRPC, did not achieve better classification accuracy. The reason is that NG-RPC, which uses only spectral features, ignores the importance of spatial structure information; OGRPC, which uses only the last layer of convolution features and spatial features, ignores the more powerful spectral features of the shallow layer. PGRPC has not achieved better classification accuracy. The reason is that PCA often fails to show good class discrimination when processing high-dimensional data.

**Table 7:** The classification results (OA (%) and Kappa (%)) obtained by using GRPC and its three variants.

Data set	GRPC		NG-RPC		OGRPC		PGRPC	
	OA	Kappa	OA	Kappa	OA	Kappa	OA	Kappa
Indian Pines	98.09 ± 0.32	97.78 ± 0.37	95.78 ± 0.19	95.27 ± 0.21	91.67 ± 0.28	89.08 ± 0.17	96.59 ± 0.26	96.04 ± 0.31
Pavia University	99.64 ± 0.14	99.51 ± 0.20	98.12 ± 0.12	98.05 ± 0.14	96.67 ± 0.58	96.28 ± 0.57	98.86 ± 0.29	98.45 ± 0.39
Kennedy Space Center	96.53 ± 0.75	96.12 ± 0.87	93.80 ± 0.42	92.71 ± 0.16	90.59 ± 0.25	89.41 ± 1.39	95.27 ± 0.17	94.73 ± 0.19

The time cost of the algorithm is one of the important factors affecting its application in remote sensing. This section evaluates the time consumption of the proposed GRPC and existing HSI classification methods such as SAE, 3D-CNN, Gabor-Based and RPNet. As shown in Table 8, the methods based on SAE and 3D-CNN are the slowest, because these two methods need to be pretrained first, and then fine-tuned to achieve the convergence strategy; compared with SAE and 3D-CNN, Gabor-Based runs relatively faster without pretraining operations, but Gabor-Based reduces its efficiency when extracting each high-dimensional pixel feature; For GRPC and RPNet, the RPNet method is obviously faster, because GRPC is affected by the extraction of spatial features and the fusion of spectral and spatial features. This process occupies one third of the total running time of the method. However, GRPC classification accuracy is higher, and the overall time consumption is acceptable. All the experimental environments here remain unchanged. For the computing environment and parameters used in this section, please refer to the beginning of this section.

**Table 8:** The computation time using five methods on three data sets (s).

Data set	SAE	3D-CNN	Gabor-Based	RPNet	Ours
Indian Pines	316.32	279.74	131.17	20.18	31.62
Pavia University	603.39	574.63	192.77	95.82	125.82
Kennedy Space Center	257.72	225.28	154.21	42.43	62.43

In summary, the comparison of experiments and algorithms on the three data sets shows that the GRPC method can extract highly distinguished features by combining multi-scale and multi-layer convolution information, and fuse feature information. For HSI classification, GRPC is a competitive and robust method. Specifically, the experiments in this article show that both random patches convolution and two-dimensional Gabor filters are reliable technologies, and GRPC is still robust even with limited training samples.

## Conclusions

Aiming at the problem of low HSI training samples and low classification accuracy, this paper proposes a HSI classification method GRPC that combines spatial feature information and spectral feature information. This method first performs PCA and LDA dimensionality reduction processing on HSI, which retains useful information and increases computational efficiency. Secondly, in order to effectively use the spectral and spatial information, Gabor filter and random patches convolution are used to extract the spatial and spectral features of HSI respectively. Finally, the feature stacking method is used to fuse spatial spectral information, and SVM is used to classify the image. Through experiments, several important parameters that affect the accuracy of the network are discussed separately, and through comparative experiments on the three data sets of Indian Pine, Pavia University and Kennedy Space Center, the superiority of the network is verified. Compared with the method, the OA and Kappa coefficients are improved to different degrees, and this network still has better classification accuracy even with limited training samples. In the subsequent research work, it is planned to further optimize the operating efficiency of the algorithm while ensuring the classification accuracy.

## Data Availability

The three datasets of Indian Pines, Pavia University, and Kennedy Space Center used in this study are publicly available at http://www.ehu.eus/ccwintco/index.php/Hyperspectral_Remote_Sensing_Scenes.
